# Infectious bursal disease virus suppresses humoral immunity via blocking antibody affinity maturation in chickens

**DOI:** 10.1016/j.isci.2026.115596

**Published:** 2026-04-03

**Authors:** Yuxin Guo, Zhaocan Zhou, Huixuan Jiao, Li Gao, Xiaoqi Li, Hong Cao, Yongqiang Wang, Shijun J. Zheng

**Affiliations:** 1 State Key Laboratory of Veterinary Public Health and Security, Beijing, China; 2 Animal Epidemiology of the Ministry of Agriculture, Beijing, China; 3 College of Veterinary Medicine, China Agricultural University, Beijing 100193, China

**Keywords:** Poultry medicine, Immunology, Virology

## Abstract

Blockade of antibody affinity maturation causes vaccination failure, yet whether pathogens exploit this process for immunosuppression remains poorly understood. Infectious bursal disease virus (IBDV) causes immunosuppression in chickens, but its underlying mechanism remains unclear. We show that the anti-IBDV VP2 affinity constant (Ka) remained unchanged between primary and secondary responses, indicating antibody affinity maturation failure. In contrast, anti-NDV F protein antibodies exhibited a >4-fold Ka increase upon secondary response, but this increase was abolished by IBDV coinfection. Consistently, anti-NDV HI titers were reduced in NDV and IBDV-coinfected chickens. IBDV infection decreased splenic IgM^+^ B cells and upregulated *IL2*, *IFNG*, and activation-induced cytidine deaminase (AID) expression. We identified VP5 and double-stranded RNA (dsRNA as key viral components inducing *IFNG* and *IL2*, which skews the immune response toward cellular immunity. These findings identify the blockade of antibody affinity maturation as a key mechanism of IBDV-induced immunosuppression, offering a mechanistic explanation for vaccination failure and viral immune evasion.

## Highlights

• IBDV suppresses immune response via blocking antibody affinity maturation
• IBDV and NDV coinfection inhibits the affinity maturation of anti-NDV antibodies
• IBDV and NDV coinfection reduces the HI titer of anti-NDV antibodies in serum
• IBDV VP5 and dsRNA upregulate IL-2, IFN-γ and AID expression, driving Th1 response

## Graphical abstract

**Figure:**
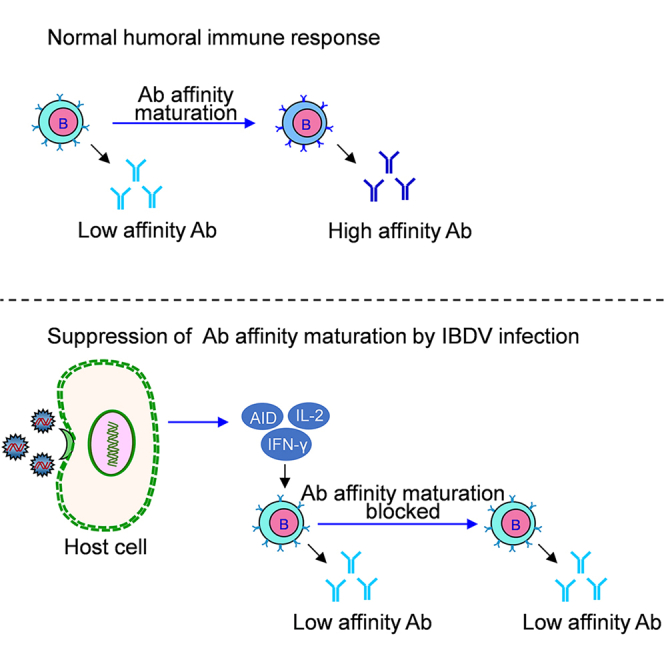

## Introduction

High-affinity neutralizing antibodies play a crucial role in protecting the host from pathogenic infection. Thus, continuous increase of antibody affinity by the affinity maturation process is one of the main features of adaptive immunity to clear antigens by antibodies with high efficiency. If the affinity maturation process is blocked, a high-affinity antibody cannot be yielded, thereby affecting the clearance of antigens by the immune system and leading to vaccination failure. Infectious bursal disease virus (IBDV) is a typical immunosuppressive avian virus targeting B lymphocytes in the bursa of Fabricius, causing severe economic losses to the poultry industry across the globe. However, the exact mechanism underlying IBDV-induced immunosuppression, in particular, blocking antibody affinity maturation, is largely unknown.

During the early stage of B-cell immune response, the antibodies exhibit low affinity, whereas high-affinity antibodies are generated through affinity maturation.^1^^,^^2^ In the germinal centers of the spleen and lymph nodes, low-affinity B lymphocytes (Ka1) undergo somatic hypermutation (SHM) and class-switch recombination, giving rise to centrocytes with varying affinities.^3^^,^^4^^,^^5^ High-affinity B cell receptors (BCRs) competitively bind antigens and, upon activation by T helper (Th) cells, differentiate into plasma cells that produce high-affinity antibodies (Ka2) and memory cells.^6^^,^^7^ In contrast, low-affinity B lymphocytes undergo apoptosis and are subsequently cleared by macrophages.^8^ Following affinity maturation, memory B cells and plasma cells exhibit higher affinity than their parental cells (Ka2 > Ka1).^9^ Therefore, comparing antibody affinity between primary and recall responses provides a functional indicator of affinity maturation efficiency.

IBDV is a non-enveloped virus with a double-stranded RNA (dsRNA) genome encoding five viral proteins (VP1-VP5).^10^^,^^11^ IBDV infection mainly causes damage to the bursa of Fabricius, a central immune organ of birds for the development and maturation of B lymphocytes,^12^^,^^13^^,^^14^ and also affects peripheral lymphoid organs such as the spleen.^15^ Very virulent strains (vvIBDV) and variant strains (varIBDV) primarily target and destroy immature IgM^+^ B lymphocytes, affecting their development and maturation.^16^^,^^17^^,^^18^ Notably, IBDV-infected chickens did not respond well to vaccination against Avian influenza (AI),^19^ Newcastle disease (ND),^20^ and infectious bronchitis (IB),^21^ causing vaccination failure, and show increased susceptibility to secondary infections.^22^ Recent emergence of varIBDV and vvIBDV in flocks further complicated avian disease prevention and control efforts.^12^^,^^23^ Previous studies have primarily attributed IBDV-induced immunosuppression to B-cell depletion and reduced antibody titers.^24^ However, whether IBDV also impairs the process of antibody affinity maturation remains elusive.

In this study, we attempted to determine the impact of IBDV infection on antibody affinity maturation by measuring Ka of serum antibodies of chicken post viral infection, and investigated the mechanism underlying the IBDV-induced suppression of antibody affinity maturation. We found that the Ka for anti-IBDV antibodies in the serum of IBDV-infected chickens did not increase in the amnestic immune response to IBDV infection, indicating that the humoral immune response failed to undergo the antibody affinity maturation process. In contrast, the Ka of serum antibodies to NDV in the secondary immune response to NDV infection markedly increased by over 4-folds compared to that of the primary immune response (Ka, 1.61×10^6^ vs. 3.43×10^5^), indicating the success of anti-NDV antibodies in affinity maturation, but the increase of Ka of serum antibodies to NDV was completely abolished by coinfection with IBDV or infection with IBDV 4 days ahead of NDV primary infection, suggesting that IBDV-infection suppresses humoral immunity by blocking antibody affinity maturation.

## Results

### IBDV infection inhibited the affinity maturation of antibodies against IBDV

**Figure 1:**
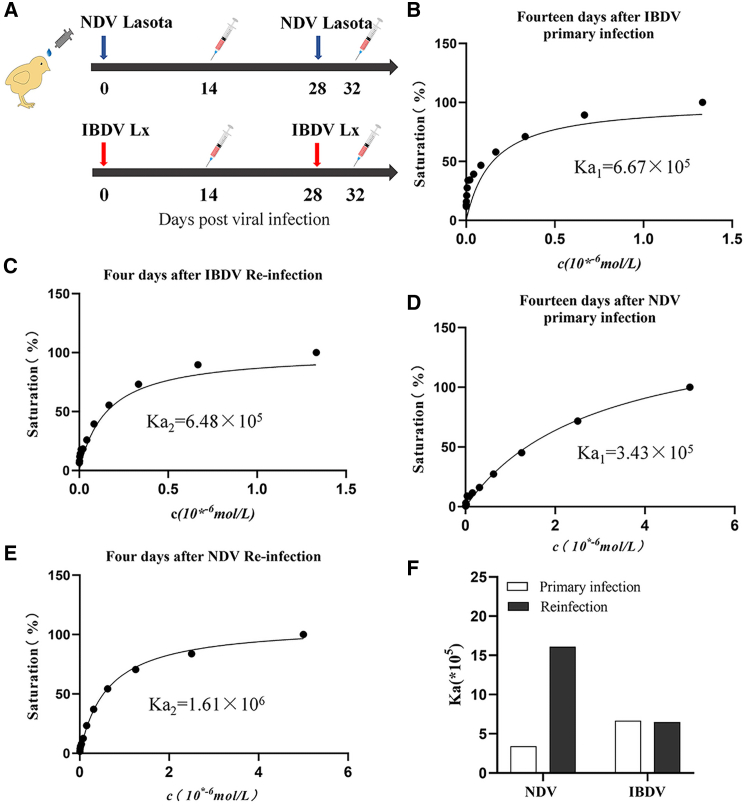
IBDV infection inhibits the affinity maturation of antibodies against viral antigens (A) Schematic diagram illustrates the timeline of viral infection and serum collection. Blue arrows indicate primary and secondary infections with IBDV Lx, and red arrows indicate primary and secondary infections with NDV LaSota. Serum samples were collected at 14 days post-primary infection and 4 days post-secondary infection (32 days post-primary infection). (B–E) Antibody affinity constants against IBDV VP2 or NDV F protein analyzed by indirect ELISA. Saturation binding curves were generated using serial dilutions of the coating antigen (IBDV VP2 or NDV F protein), with the x axis representing antigen concentration (c, in 10^−6^ mol/L). Affinity constants (Ka) were calculated from the curves. Nine-day-old SPF chickens were inoculated with NDV LaSota (10^6^ TCID_50_, 200 μL, B and C) or IBDV Lx (10^6^ TCID_50_, 200 μL, D and E) via nasal and ocular drops (*n* = 6/group). Four weeks post initial infection, chickens were challenged with homologous viral strains. Serum samples were collected 14 days post-initial viral infection (B and C) and 4 days post-secondary infection (D and E). Ka1 and Ka2 denote affinity constants after primary and secondary infection, respectively. (F) Comparison of serum antibody affinity constants between NDV- and IBDV-infected chickens. Data represent pooled serum samples from six replicates per group.

It is well-known that IBDV infection suppresses humoral immunity;^10^ however, the underlying mechanisms remain elusive. Given that antibody affinity maturation is crucial for generating high-affinity antibodies, we hypothesized that IBDV infection might interfere with this process. To test this hypothesis, we infected specific pathogen-free (SPF) chickens with IBDV and determined antibody affinity by measuring the antibody affinity constant (Ka),^25^^,^^26^ using NDV as controls. As shown in Figure 1A, 9-day-old SPF chickens were infected with either IBDV Lx or NDV LaSota strain, followed by collecting blood samples at indicated time points (14 days post initial viral inoculation and 4 days post viral reinfection) for the determination of antibody affinity constant. As shown in Figures 1B and 1C, the antibody affinity constant remained virtually unchanged upon IBDV Lx reinfection compared to the initial viral infection (Ka, 6.67×10^5^ vs. 6.48×10^5^). In contrast, the antibody affinity constant increased by 4.7-fold following NDV LaSota reinfection compared to the initial infection (Ka, 1.61×10^6^ vs. 3.43×10^5^), indicating the production of higher-affinity antibodies against NDV (Figures 1D–1F). These data suggest that the infection of chickens by IBDV fails to generate higher-affinity antibodies against IBDV via the antibody affinity maturation process in the secondary immune response.

### IBDV infection impaired anti-NDV antibody affinity maturation in chickens inoculated with NDV

**Figure 2:**
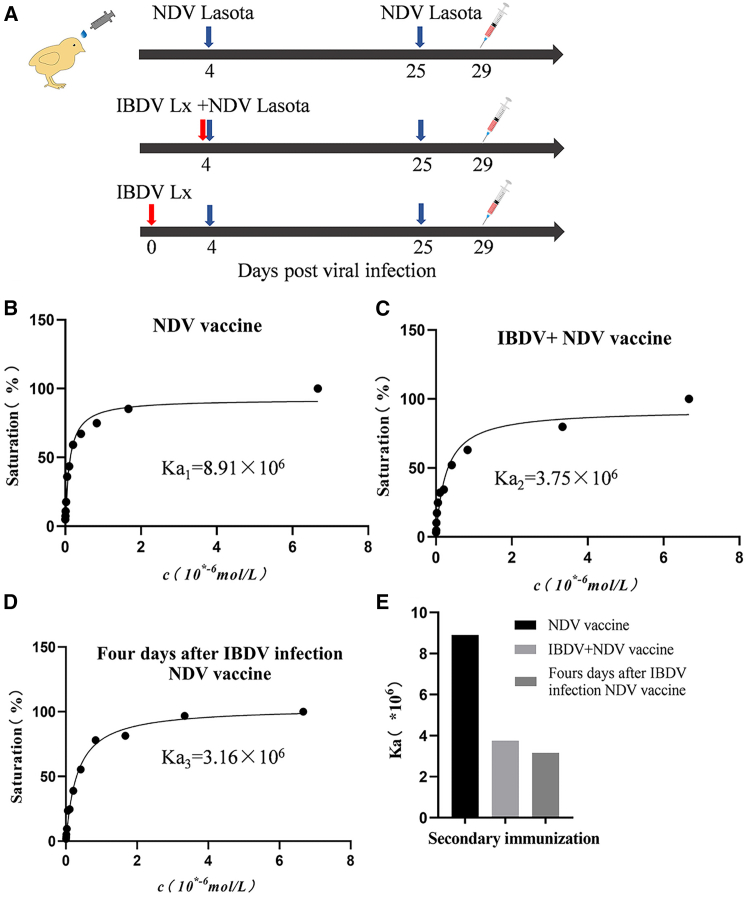
IBDV infection inhibits the affinity maturation of anti-NDV LaSota antibodies following vaccination (A) Schematics illustrate the timeline of vaccination and serum collection. Red arrows indicate immunization with NDV LaSota vaccine, blue arrows indicate infection with IBDV Lx, and the “IBDV Lx + NDV LaSota” group received both treatments at the indicated time points. (B–D) Antibody affinity constants against NDV analyzed by indirect ELISA. Nine-day-old SPF chickens were randomly divided into four groups (*n* = 15/group): NDV LaSota vaccination alone (10^6^ EID50, 100 μL/bird; B), IBDV Lx (10^6^ TCID_50_, 200 μL/bird) and NDV LaSota (10^6^ EID50, 100 μL/bird) co-inoculation (C), IBDV Lx infection (10^6^ TCID_50_, 200 μL/bird) followed by NDV LaSota vaccination 4 days post-infection (D), and PBS control. Three weeks post-initial vaccination, groups B-D received NDV LaSota boosters. Sera were collected 4 days post-booster for anti-NDV antibody affinity analysis. (E) Comparison of anti-NDV antibody affinity constants determined by indirect ELISA (B–D). The data represent pooled samples from six replicates.

As IBDV infection significantly suppressed host immune response to vaccination against other diseases in intensive poultry farming systems^10^^,^^20^ and chickens with IBDV infection failed to generate higher-affinity antibodies against IBDV in the immune response to booster immunization, we set out to determine whether the suppressive effect of IBDV infection on the efficacy of NDV vaccine was attributed to IBDV-induced failure of antibody affinity maturation. As shown in Figure 2A for the timeline of the experiment, a total of sixty 9-day-old SPF White Leghorn chickens were divided into 4 groups with 15 each. Among these chickens, three groups were infected with NDV only, NDV+IBDV, or with NDV 4 days post-IBDV infection, respectively, and the controls were treated with PBS. Three weeks post-initial viral infection, three groups of chickens initially infected with viruses were boostered with NDV vaccine. Four days post-booster immunization, blood samples were collected, and serum antibody affinity constants were determined using indirect ELISA. As a result, the concurrent infection of chickens with NDV and IBDV significantly reduced anti-NDV antibody affinity constants compared to that of the NDV vaccinated-only group (Ka, 3.75 х10^6^ vs. 8.91×10^6^) (Figures 2B–2E), and so did the infection of chickens with IBDV 4 days ahead of NDV infection (Ka, 3.16 х10^6^ vs. 8.91×10^6^). These data directly demonstrate that IBDV infection inhibited the generation of high-affinity anti-NDV antibodies in the secondary immune response of chickens to NDV booster immunization, indicating that IBDV infection impaired antibody affinity maturation in the immune response to heterologous antigens.

### IBDV infection significantly suppresses HI titers of antibodies against NDV

**Figure 3:**
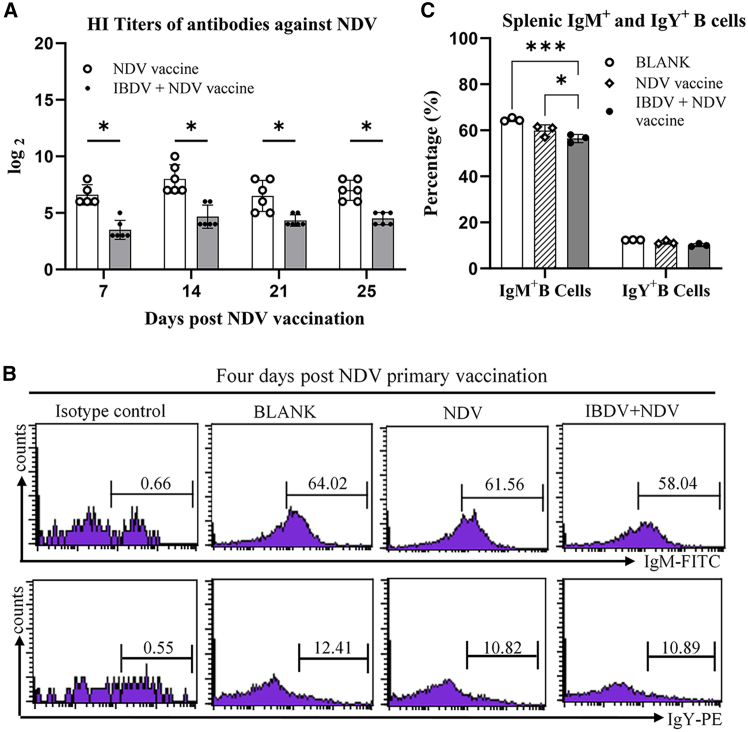
IBDV infection reduces HI titers and splenic IgM^+^ B-cell proportion post-NDV vaccination (A) HI titers of antibody against NDV LaSota were determined by hemagglutination inhibition assay. (B and C) Quantification of IgM^+^ and IgY^+^ splenic B lymphocytes by flow cytometry at 4 days post-infection. Splenic lymphocytes were isolated and dual-stained with IgM/IgY and Bu-1b antibodies. Data are representative of six independent biological replicates and are expressed as mean ± SD. Comparisons between groups were conducted using Student’s *t* test. ∗∗∗, *p* < 0.001 and ∗, *p* < 0.05.

As B cells are responsible for humoral immunity, and we found that IBDV infection impairs antibody affinity maturation, it was intriguing to determine the impact of IBDV infection on the levels of specific antibodies against other pathogens using NDV as a model and on the proportion of IgM^+^ and IgY^+^ B lymphocytes in the spleen of chickens following NDV vaccination. Thus, we infected 9-day-old SPF chickens with IBDV, meanwhile inoculated chickens with NDV LaSota vaccine as described above in Figure 2A, and examined the levels of antibodies against NDV using HI assay and the proportion of IgM^+^ and IgY^+^ B lymphocytes using flow cytometry. As shown in Figure 3A, the anti-NDV antibody titers in the serum of chickens infected with NDV plus IBDV were significantly lower than those of chickens merely infected with NDV 7, 14, 21, and 25 days post initial inoculation, indicating that IBDV infection severely affected primary humoral immune response to NDV infection.

**Figure 4:**
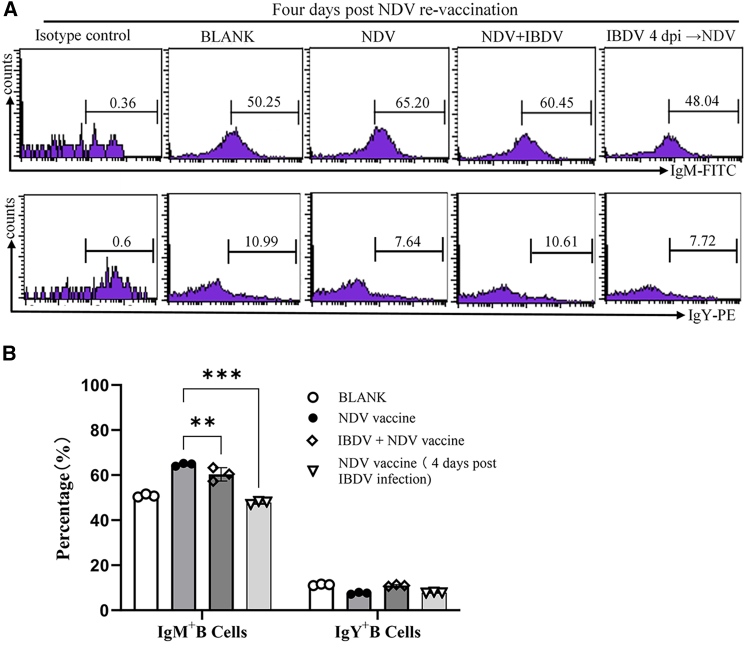
IBDV infection reduces splenic IgM^+^ B-cell proportion post-NDV reinfection (A and B) Splenic lymphocytes isolated 4 dpi from virus-inoculated chickens were triple-stained with anti-IgM-FITC, IgY-PE, and Bu-1b-AF647 antibodies for flow cytometry analysis. (A) Representative flow cytometry plots. (B) Statistical analysis of IgM^+^ and IgY^+^ B-cell percentages. Data represent mean ± SD from six independent replicates. Comparisons between groups were conducted using Student’s *t* test. ∗∗∗, *p* < 0.001; ∗∗, *p* < 0.01; and ∗, *p* < 0.05.

To investigate the mechanisms underlying the IBDV-induced suppression of serum antibody affinity and titers, we performed flow cytometry to assess the proportions of IgM^+^ and IgY^+^ B lymphocytes in the spleen using flow cytometry, which are directly associated with antibody production. As a result, the proportion of IgM^+^ but not IgY^+^ B lymphocytes in the spleen of chickens with NDV-IBDV infections was significantly lower than that of chickens merely infected with NDV 4 days post initial inoculation (*p* < 0.05) (Figures 3B and 3C), suggesting that IBDV infection mainly affects naive B lymphocytes rather than memory B cells in the initial immune response. To further strengthen the results, we inoculated chickens with NDV or NDV plus IBDV, and examined the proportions of IgM^+^ and IgY^+^ B lymphocytes in the spleen 4 days post-NDV booster vaccination. As shown in Figures 4A and 4B, the proportions of splenic IgM^+^ B lymphocytes in chickens that were initially infected with NDV-IBDV or with IBDV 4 days ahead of NDV vaccination were markedly lower than those of those initially inoculated with NDV only. These data suggest that IBDV infection compromises host immunity by depleting splenic IgM^+^ B lymphocytes, thereby reducing the production of specific antibodies.

### IBDV infection preferentially enhanced Th1 cytokine expression and activation-induced cytidine deaminase (AID) expression

**Figure 5:**
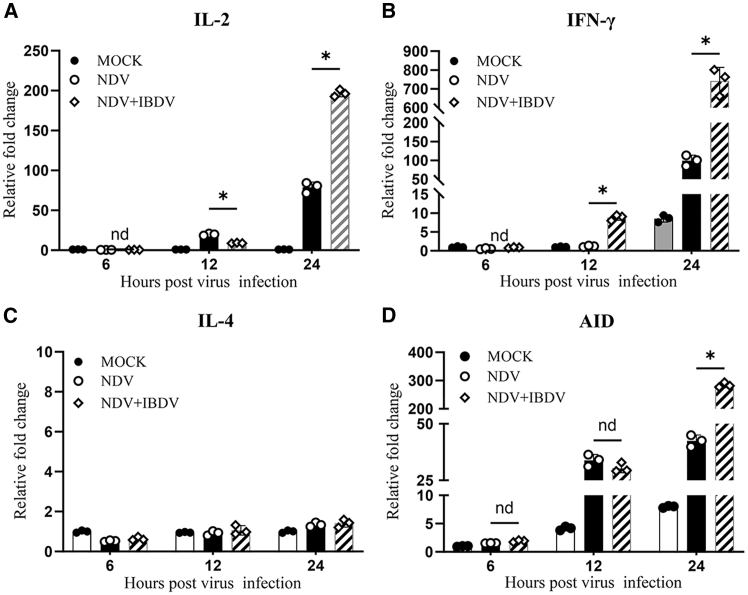
IBDV infection upregulates the expression of Th1 cytokines and cytidine deaminase (AID) in DT40 cells (A–D) The mRNA expression of cytokines and *AID* in DT40 cells with or without NDV or NDV-IBDV infection. DT40 cells were mock-infected, infected with NDV LaSota, or co-infected with NDV LaSota and IBDV Lx (MOI = 0.5). At different time points (6, 12, and 24 h) post-infection, cells were harvested and examined for the mRNA expression of *IL2* (A), *IFNG* (B), *IL4* (C), and *AID* (D) by RT-PCR. Data are presented as mean ± SD from three independent replicates. Comparisons between groups were conducted using one-way ANOVA. ∗, *p* < 0.05; nd, *p* > 0.05.

As Th1 cytokines are known to promote cell-mediated immune response while inhibiting antibody affinity maturation,^27^ we analyzed the mRNA expression of affinity maturation-related genes (*IL2*, *IL4*, *IFNG,* and cytidine deaminase) in DT40 cells and in the splenic tissues of chickens with NDV infection in the presence or absence of IBDV using RT-qPCR. Our data show that the mRNA expressions of *IL2* and *IFNG*, but not *IL4*, in DT40 cells with NDV-IBDV infection markedly increased compared to those with mock or NDV infection only (Figures 5A–5C), indicating that IBDV infection preferentially enhanced Th1 cytokine expression. Furthermore, as activation-induced cytidine deaminase (AID) serves as an enzyme that induces SHM, antibody class switching, and regulates the production of antibodies,^28^ we examined the expression of *AID* in DT40 cells with NDV infection in the presence or absence of IBDV. As a result, *AID* expression markedly increased compared to that of those with mock or NDV-infection only (Figure 5D). These data suggest that the IBDV-induced upregulation of Th1 cytokines (*IL2* and *IFNG*) may directly contribute to the suppression of humoral immunity.

To strengthen these results, we infected chickens with NDV concurrently with IBDV or with IBDV 4 days in advance, and examined the mRNA expressions of *IL2*, *IL4*, *IFNG*, and *AID* in the spleen of chickens with NDV infection or NDV-IBDV coinfection. As a result, the expressions of *IL2*, *IFNG*, and AID in the spleen of chickens with NDV infection expressed Th1 cytokines and *AID.*

**Figure 6:**
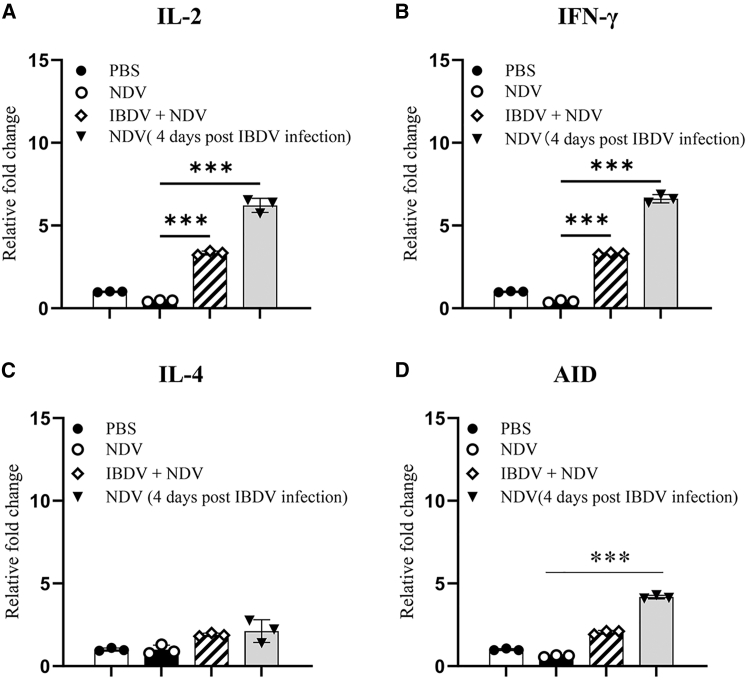
IBDV infection upregulates splenic Th1 cytokines and AID expression in IBDV-NDV coinfected chickens (A–D) Transcript levels of *IL2* (A), *IFNG* (B), *IL4* (C), and *AID* (D) in splenic tissue were analyzed by RT-qPCR 12 h post-NDV LaSota revaccination. mRNA expression changes were normalized to mock-infected controls. Data are presented as mean ± SD from three independent replicates. Comparisons between groups were conducted using Student’s *t* test. ∗∗∗, *p* < 0.001.

Expression in the presence of IBDV was remarkably greater than that of chickens with IBDV infection only, whereas *IL4* expression was unaffected by IBDV infection (Figures 6A–6D), confirming that IBDV infection upregulated expressions of Th1 cytokines that might be directly involved in the suppression of humoral immunity.

### IBDV genomic dsRNA and VP5 are involved in upregulating the expression of factors associated with affinity maturation in DT40 cells

**Figure 7:**
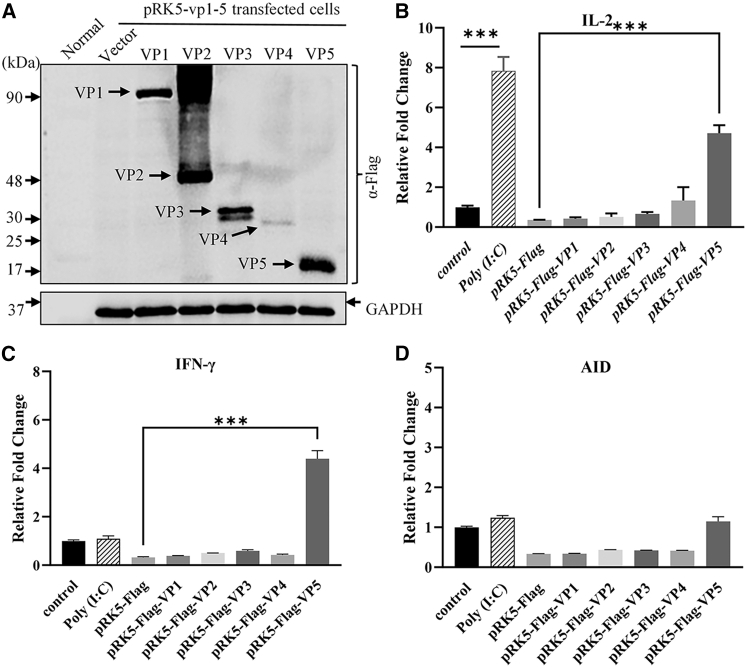
Effect of IBDV viral proteins and dsRNA on Th1 cytokine and AID expression in DT40 cells (A) IBDV viral protein expression analysis by western blot. DT40 cells were transfected with 5 μg of pRK5-Flag, pRK5-VP1, pRK5-VP2, pRK5-VP3, pRK5-VP4, or pRK5-VP5. Cells were harvested 24 h post-electroporation, and lysates were probed with anti-Flag and anti-GAPDH antibodies. Endogenous *GAPDH* served as the loading control. (B–D) qRT-PCR analysis of *IL2* (B), *IFNG* (C), and *AID* (D) mRNA levels in DT40 cells transfected with 5 μg pRK5-Flag, pRK5-VP1-VP5 plasmids, or poly(I:C). Total RNA was extracted 24 h post-transfection. Data are presented as mean ± SD from three independent replicates. Comparisons between groups were conducted using one-way ANOVA. ∗∗∗, *p* < 0.001.

As IBDV infection upregulated the expression of Th1 cytokines, it was logical to determine which IBDV components (VP1-VP5 proteins and viral genomic RNA) upregulated the expression of *IL2*, *IFNG*, and *AID* in host cells. We transfected DT40 cells with 5 μg/mL eukaryotic expression vectors encoding IBDV viral proteins (VP1-VP5) or poly(I:C), a synthetic analog of dsRNA, via electroporation, and examined the expression levels of *IL2*, *IFNG*, and *AID* by qRT-PCR. As shown in Figure 7A, ectopic expressions of VP1-VP5 could be well detected in cells 20 h post-transfection with pRK5-VP1-5. Interestingly, the transfection of DT40 cells with pRK5-Flag-vp5 or poly(I:C) significantly increased *IL2* expression compared to that of controls (Figure 7B). Furthermore, the ectopic expression of VP5 remarkably upregulated expressions of *IFNG* and *AID* (Figures 7C and 7D). These findings indicate that both the viral protein VP5 and dsRNA of IBDV were involved in upregulating the expression of Th1 cytokines and AID.

Taken together, our data show that IBDV infection compromised humoral immune response, during which antibody affinity failed to increase in the secondary immune response, suggesting that IBDV infection affected antibody affinity maturation. Coinfection of chickens with IBDV and NDV also affected the antibody affinity maturation of humoral immune response to NDV infection, and resulted in failure of antibody affinity increase in amnestic immune response, suggesting that IBDV-induced failure of antibody affinity maturation is nonspecific. Furthermore, IBDV infection upregulated the expressions of *IL2*, *IFNG*, and *AID* in the spleen of chickens without affecting *IL4*, thereby suppressing humoral immune response. Importantly, our data show that VP5 and dsRNA of IBDV were involved in upregulating the expression of Th1 cytokines and AID. Thus, IBDV infection inhibits antibody affinity maturation by VP5-and viral genome-induced expressions of Th1 cytokines and *AID*, consequently suppressing humoral immune response and leading to secondary infections and vaccination failure.

## Discussion

High-affinity antibodies are generated via one or more rounds of the antibody affinity maturation process during the recall immune response, and high-affinity antibodies play a crucial role in protecting the host from pathogenic infection. Thus, continuous increase of antibody affinity by the affinity maturation process is one of the main features of adaptive immunity to clear antigens with high efficiency. If the affinity maturation process is blocked, a high-affinity antibody cannot be yielded, thereby affecting the clearance of antigens by the immune system and even leading to vaccination failure. It is well established that an immunosuppressive pathogen compromises host immunity by targeting immune cells in favor of its replication and survival. However, whether a pathogen, such as IBDV, a typical immunosuppressive virus for chicken, suppresses host immune response by affecting antibody affinity maturation process is currently unknown. In the present study, first, our data show that IBDV infection inhibited the affinity maturation of antibodies against IBDV. Second, IBDV infection impaired anti-NDV antibody affinity maturation and significantly suppressed HI titers of antibodies against NDV, suggesting that IBDV-induced failure of antibody affinity maturation is non-specific. Third, IBDV infection preferentially enhanced Th1 cytokine (*IL2*, *IFNG*) and *AID* expressions, which are favorable to drive immune response toward cellular immunity. Importantly, our data show that IBDV genomic dsRNA and VP5 are involved in upregulating the expression of factors (*IL2*, *IFNG*, *AID*) in DT40 cells that are associated with suppressing affinity maturation. Thus, IBDV infection inhibits antibody affinity maturation by VP5-or viral genome(dsRNA)-induced expressions of Th1 cytokines and *AID*, thereby suppressing humoral immune response. Using the infection of chickens by IBDV as a model, we show that the viral inhibition of antibody affinity maturation causes host immunosuppression, defining a mechanism of pathogenic suppression of the humoral immune response.

IBDV, an immunosuppressive virus targeting B lymphocytes in the bursa of Fabricius in chickens, causes lymphocyte depletion in immune organs and immunosuppression in chickens, leading to considerable economic losses to the poultry industry across the globe.^10^ Previous studies demonstrated that bursal lymphocyte depletion in infected chickens recovered approximately one week post-infection, accompanied by the formation of reorganized large follicles and small follicles with corticomedullary dysplasia, and these follicles predominantly harbor undifferentiated follicular B lymphocytes, which fail to generate mature B cells.^29^^,^^30^ The affinity constant of antibodies reflects their binding capacity to antigenic epitopes, with higher values indicating stronger antigen-antibody interactions.^9^ In this study, SPF chicks were primarily infected with IBDV Lx strain, followed by secondary infection with the same virus, using NDV infection as parallel controls. Surprisingly, the affinity constant of antibodies against IBDV did not increase post-secondary infection with IBDV, whereas the affinity constant of antibodies against NDV increased by 4.7-folds following NDV LaSota reinfection compared to the primary infection, suggesting that chickens with IBDV infection fail in antibody affinity maturation to generate higher-affinity antibodies against IBDV in secondary immune response. As a consequence, the anti-IBDV antibodies did not have sufficient affinity to bind to IBDV for neutralization, thereby compromising subsequent viral clearance. These findings highlight a mechanism by which IBDV subverts humoral immunity, extending beyond quantitative antibody reduction to the qualitative impairment of antibody functionality.

In intensive poultry rearing systems, IBDV infection compromises the immune response of chickens that are expected to maintain high vaccine-induced antibody titers. For instance, prior exposure to novel variant IBDV significantly reduces HI antibody titers against NDV LaSota strain, thereby diminishing vaccine efficacy.^20^ Consistently, our data reveal that the infection of chickens with IBDV suppressed the affinity maturation of antibodies against NDV, regardless of whether IBDV infection occurs concurrently with or prior to NDV vaccination. Additionally, chickens with a history of IBDV infection exhibited heightened susceptibility to AIV, potentially facilitating the introduction of AIV into flocks, posing a threat to public health.^31^ Thus, the persistent circulation of IBDV in the environment may induce prolonged immunosuppression in chickens, impairing the capacity of chickens to generate a diverse, high-affinity immunoglobulin repertoire against pathogens such as NDV,^19^ AIV,^20^ and IBV.^21^ This underscores a critical challenge in poultry health management, because IBDV-induced compromised humoral immunity not only undermines vaccination efficacy but also exacerbates the risk of secondary infections.

It was reported that IBDV infection suppressed the humoral immune response of chickens to NDV,^19^ AIV,^20^ and IBV^21^ infections. Besides the bursal damage caused by IBDV infection, the mechanism underlying IBDV-induced immunosuppression in chickens is largely unknown. Our data show that the affinity constant (Ka) of antibodies against NDV significantly increased in the secondary immune response to NDV reinfection, but this increase was abolished by IBDV infection. In other words, IBDV infection resulted in a significant reduction in the affinity constant of antibodies against NDV in chickens with NDV LaSota inoculation, which explained that IBDV infection suppressed the specific antibody response to vaccination against other pathogenic infections. This may also be the cause of immunization failure in the control of avian diseases on poultry farms. Furthermore, consistent with previous studies, IBDV infection reduces anti-NDV HI antibody titers for over 3 weeks after ND vaccination (Figure 3A). These observations demonstrate that IBDV infection induces persistent immunosuppression, leading to the sustained impairment of humoral immunity. This prolonged immune dysfunction underscores the challenge of achieving effective vaccine protection in IBDV-exposed poultry populations.

During avian embryonic development, B-lymphocyte precursors migrate from the bone marrow to the bursa of Fabricius for development and maturation. Mature B lymphocytes subsequently migrate to peripheral lymphoid organs, such as the spleen and tertiary lymphoid tissues. The spleen harbors a substantial population of mature B lymphocytes, which are critical for antibody production and secretion, and serves as a primary organ for antibody affinity maturation^32^^,^^33^. Previous studies have demonstrated that surface immunoglobulin M (sIgM)-bearing B lymphocytes are the primary targets of IBDV in the bursa of Fabricius, with viral infection causing severe destruction of IgM^+^ B cells. Notably, the sIgM λ light chain appears to facilitate IBDV attachment and infection.^16^^,^^17^ In this study, the proportions of IgM^+^ B lymphocytes in the spleen were significantly reduced in chickens during the acute phase of IBDV infection, followed by NDV LaSota vaccination, indicating the early destruction of IgM^+^ B lymphocytes by IBDV infection (Figures 3B and 3C). Furthermore, even three weeks post-IBDV infection, the proportion of IgM^+^ B lymphocytes in the spleen was much lower than that of chickens immunized with ND vaccination alone (Figures 4A and 4B), suggesting that IBDV-induced damage to splenic IgM^+^ B lymphocytes persists into the subclinical phase. The reduced population of IgM^+^ B lymphocytes in the spleen may impair antibody class switching, which could partially explain the observed suppression of ND vaccine-induced antibody affinity maturation. However, the exact mechanism requires further investigation.

IL-4 is an immunomodulatory cytokine primarily secreted by Th2 cells and Tfh cells, promoting antibody class switching and subsequent affinity maturation.^34^ In contrast, IFN-γ antagonizes IL-4 activity, driving robust cell-mediated immunity. IFN-γ can suppress the IL-4-dependent production of IgE and IgG1, thereby modulating humoral responses.^27^ IL-2, which stimulates T cell proliferation, is critical for Th2 polarization *in vivo* but suppresses Tfh cell differentiation, a process essential for regulating B cell responses and germinal center formation.^35^ AID, a cytidine deaminase, contributes to antibody affinity maturation by initiating SHM and class switch recombination (CSR), both processes collectively driving the production of high-affinity antibodies.^32^^,^^33^^,^^36^ In this study, the increase of AID expression following IBDV infection, both *in vivo* and *in vitro,* was quite unexpected, suggesting that the increase in AID expression may not contribute directly to antibody affinity maturation in the scenario of IBDV infection. Of note, as the avian immune system varies considerably from that of mammals, the exact role of AID in chicken antibody maturation needs to be determined. Interestingly, our data show that IBDV-infection upregulated the mRNA expression of *IL2* and *IFNG*, while *IL4* was unaffected, indicating that IBDV infection mainly induced Th1 cytokine expression that drives immune response toward cell-mediated immunity, meanwhile suppressing humoral immune response. Our prior data show that IBDV VP5 acts as a virulence factor responsible for IBDV-induced apoptosis by interacting with voltage-dependent anion channel 2 (VDAC2) and receptor for activated protein kinase C1 (RACK1) in mitochondria.^37^^,^^38^ In this study, the ectopic expression of VP5 upregulated the mRNA expression of *IL2* and *IFNG* in DT40 cells, suggesting a previously unrecognized role of VP5 in regulating immune response. Recently, we found that IBDV genomic dsRNA triggered pyroptosis in host cells via the activation of caspases that cleaved chicken gasdermin E (chGSDME).^39^ In this study, we found that VP5 and poly(I:C) upregulated the expression of *IL2* and *IFNG* in DT40 cell lines, the two cytokines that negatively regulate affinity maturation. These findings indicate that the IBDV-mediated suppression of antibody affinity maturation is primarily driven by the viral protein VP5 and its double-stranded genomic RNA, which skew the immune response toward the activation of cell-mediated pathways at the expense of humoral immune response efficacy.

In summary, our data show that IBDV infection compromised humoral immune response, during which antibody affinity failed to increase in the secondary immune response, suggesting that IBDV infection affected antibody affinity maturation. Infection of chickens with IBDV also affected the antibody affinity maturation of humoral immune response to NDV infection, and resulted in failure of antibody affinity increase in amnestic immune response, suggesting that IBDV-induced failure of antibody affinity maturation is nonspecific. Furthermore, IBDV infection markedly reduced the proportion of splenic IgM^+^ B lymphocytes, but upregulated the expressions of *IL2*, *IFNG*, and *AID* in chickens without affecting *IL4*, thereby suppressing humoral immune response. This consequence is mechanistically driven by the viral protein VP5 and dsRNA. These findings provide insight into the mechanisms underlying IBDV-induced immunosuppression and the frequent vaccination failures in the prevention and control of avian diseases on poultry farms, offering potential targets for improving vaccine strategies and disease management.

### Limitations of the study

This study has several limitations. First, antibody affinity constants were determined using pooled sera due to limited sample volume and the requirement for sufficient purified antibody to generate complete saturation curves. As a result, inter-individual variability could not be assessed, and statistical comparison of Ka values was not feasible. Second, the ELISA-based affinity estimation provides an apparent equilibrium affinity derived from polyclonal sera but does not resolve real-time binding kinetics, as would be achieved using biophysical methods such as surface plasmon resonance (SPR) or biolayer interferometry (BLI). Finally, although our data demonstrate that IBDV blocks affinity maturation, the precise quantitative contribution of affinity impairment relative to B-cell depletion in overall immunosuppression remains to be determined. Future studies incorporating individual-level analysis and kinetic binding assays will further refine these findings.

## Resource availability

### Lead contact

Further information and requests for resources and reagents should be directed to and will be fulfilled by the Lead Contact, Shijun J. Zheng (sjzheng@cau.edu.cn).

### Materials availability

The new plasmids generated in this study have not been deposited in a public repository but are available from the lead contact upon reasonable request.

### Data and code availability

Data reported in this paper will be shared by the lead contact upon request. This paper does not report original code. Any additional information required to reanalyze the data reported in this paper is available from the lead contact upon request.

## Acknowledgments

We thank Prof. Jue Liu from the Yangzhou University for his assistance. This work was supported by the 10.13039/501100012166National Key Research and Development Program of China (2022YFD1800300) and 10.13039/501100010038the earmarked fund for the China Agriculture Research System (CARS-40).

## Author contributions

Y.G. and Z.Z.: investigation, data curation, methodology, formal analysis, visualization, and writing – original draft. Y.G. and Z.Z. contributed equally to this work. H.J.: investigation, data curation, validation, formal analysis, and writing – review and editing. Y.W.: supervision, project administration, methodology, investigation, validation, resources, and writing – review and editing. S.J.Z.: conceptualization, supervision, project administration, funding acquisition, resources, and writing – review and editing. L.G., X.L., and H.C.: investigation, resources, and validation. Y.W. and S.J.Z. are co-corresponding authors. All authors read and approved the final manuscript.

## Declaration of interests

The authors declare no competing interests.

## STAR★Methods

### Key resources table

**Table:** 

REAGENT or RESOURCE	SOURCE	IDENTIFIER
**Antibodies**		

Mouse monoclonal anti-Flag	Sigma-Aldrich	Cat#B3111; RRID: AB_2910145
Mouse monoclonal anti-IBDV VP1–VP5	This paper	N/A
HRP-conjugated goat anti-chicken IgG (H&L)	Bioss	Cat#bs-0310G-HRP; RRID: AB_10894173
HRP-conjugated goat anti-mouse IgG (H&L)	Dingguo Changsheng Biotechnology	Cat#SH-0011
Mouse anti-chicken IgM-FITC (clone M-1)	SouthernBiotech	Cat#8310-02; RRID: AB_2796489
Mouse anti-chicken IgY-PE (clone G-1)	SouthernBiotech	Cat#8320-09; RRID: AB_2796498
Mouse anti-chicken Bu-1-AF647 (clone AV20)	SouthernBiotech	Cat#8395-31; RRID: AB_2796547
Mouse IgG1-PE (15H6)	SouthernBiotech	Cat#0102-09, RRID: AB_2793850
Mouse IgG2b-FITC (A-1)	SouthernBiotech	Cat#0104-02; RRID: AB_2793883
Mouse IgG1-AF647(15H6)	SouthernBiotech	Cat#0102-31; RRID: AB_2793863

**Bacterial and virus strains**		

Infectious bursal disease virus (IBDV), Lx strain	Laboratory of Jue Liu	N/A
Newcastle disease virus (NDV), LaSota strain	Laboratory of Guozhong Zhang	N/A
NDV LaSota vaccine	Harbin Pharmaceutical Group Bio-Vaccine Co., Ltd	N/A

**Biological samples**		

Chicken serum samples	This paper	N/A
Chicken spleen tissues	This paper	N/A

**Chemicals, peptides, and recombinant proteins**		

Poly(I:C)	Sigma-Aldrich	Cat#P9582
Protease inhibitor cocktail (100×)	MCE	Cat#HY-K0010
IBDV VP2 protein	This paper	N/A
NDV F protein	This paper	N/A

**Critical commercial assays**		

Chicken IgM ELISA Kit	Abcam	Cat#ab157692
Chicken IgY ELISA Kit	Abcam	Cat#ab157693
EASYspin Tissue/Cell RNA Rapid Extraction Kit	Aidlab	Cat#RN2801
HiScript IV All-in-One RT SuperMix	Vazyme	Cat#R433-01
Taq Pro Universal SYBR qPCR Master Mix	Vazyme	Cat#Q712
Endotoxin-free Plasmid Maxi Kit	QIAGEN	Cat#12362

**Experimental models: Cell lines**		

Chicken: DT40 cell line (bursal lymphoma)	ATCC	Cat#CRL-2111; RRID: CVCL_0249

**Experimental models: Organisms/strains**		

Chicken: White Leghorn SPF (9-day-old)	Boehringer Ingelheim Vital Biotechnology	N/A

**Oligonucleotides**		

Primer for *IL2* Forward (5′-AGAGTCTTACGGGTCTAAATCACAC-3′)	This paper	N/A
Primer for *IL2* Revers (5′-CTCACAAAGTTGGTCAGTTCATGG-3′)	This paper	N/A
Primer for *IL4* Forward (5′-GCTCTTATGCAAAGCCTCCACAA-3′)	This paper	N/A
Primer for *IL4* Reverse (5′-TGCTGCTGGCATTCAGGAGC-3′)	This paper	N/A
Primer for *IFNG* Forward (5′-ATCATACTGAGCCAGATTGTTTCG-3′)	This paper	N/A
Primer for *IFNG* Reverse (5′-TCTTTCACCTTCTTCACGCCAT-3′)	This paper	N/A
Primer for *AID* Forward (5′-ACCGCATCACATGGTTCACCTC-3′)	This paper	N/A
Primer for *AID* Reverse (5′-TTTGGGTAGGCACGAAGGAAGTC-3′)	This paper	N/A
Primer for *GAPDH* Forward (5′-TGCCATCACAGCCACACAGAAG-3′)	This paper	N/A
Primer for *GAPDH* Reverse (5′-ACTTTCCCCACAGCCTTAGCAG-3′)	This paper	N/A

**Recombinant DNA**		

pRK5-Flag-VP1	This paper	N/A
pRK5-Flag-VP2	This paper	N/A
pRK5-Flag-VP3	This paper	N/A
pRK5-Flag-VP4	This paper	N/A
pRK5-Flag-VP5	This paper	N/A

**Software and algorithms**		

GraphPad Prism 9	GraphPad Software	https://www.graphpad.com
FlowJo v10	BD Biosciences	https://www.flowjo.com/

**Other**		

Flow cytometer	BD Biosciences	FACSCaliburTM
Quantitative PCR system	Roche	Light Cycler 480Ⅱ
Electroporator	BEX Co.	X-Porator H1

### Experimental model and study participant details

#### Cell lines

The chicken DT40 cell line (bursal lymphoma cell line; ATCC, Cat#CRL-2111) was cultured in RPMI 1640 medium supplemented with 10% fetal bovine serum at 37°C in a humidified incubator with 5% CO_2_. The sex of the cell line is not defined. The cell line was not further authenticated beyond the supplier’s characterization. The cells were routinely tested for mycoplasma contamination using a PCR-based detection method and were confirmed to be negative.

#### Viruses and plasmids

The Lx strain of infectious bursal disease virus (IBDV) was obtained from the laboratory of Jue Liu (Beijing Academy of Agriculture and Forestry, China). The LaSota strain of Newcastle disease virus (NDV) was obtained from the laboratory of Guozhong Zhang (China Agricultural University, China). The NDV LaSota vaccine was purchased from Harbin Pharmaceutical Group Bio-Vaccine Co., Ltd.

Plasmids pRK5-Flag-VP1, pRK5-Flag-VP2, pRK5-Flag-VP3, pRK5-Flag-VP4, and pRK5-Flag-VP5 were generated in this study. Plasmid DNA used for transfection was prepared using an Endotoxin-free Plasmid Maxi Kit (QIAGEN, Cat#12362).

#### Animals

Nine-day-old specific-pathogen-free (SPF) White Leghorn chickens were purchased from Boehringer Ingelheim Vital Biotechnology. Chickens were housed in individual isolators under controlled environmental conditions with free access to food and water. Animals were randomly assigned to experimental groups. Sex and weight were not stratified due to lack of labeling at purchase. All animal experiments were conducted in an ABSL-2 facility. Experimental protocols were approved by the Ethical Inspection Committee of Laboratory Animal Welfare and Animal Experiment at China Agricultural University (Approval ID: Aw50903202-2-5), and conducted in accordance with institutional and national guidelines.

### Method details

#### IBDV infection assay

Nine-day-old SPF chickens were randomly divided into two groups (n = 6 per group). Birds were inoculated via ocular and intranasal routes with NDV LaSota or IBDV Lx (10^6^ TCID_50_, 200 μL per bird). At 28 days post-infection (dpi), chickens were re-challenged with the same dose and strain. Blood samples were collected at 14 dpi and 32 dpi. Serum antibody affinity constants were determined by ELISA.

#### NDV response under IBDV infection

A total of 60 nine-day-old SPF White Leghorn chickens were randomly assigned into four groups (n = 15 per group). Chickens received either co-inoculation of IBDV Lx (10^6^ TCID_50_, 200 μL, ocular and intranasal) and NDV LaSota vaccine (10^6^ EID_50_, 100 μL, oral), IBDV followed by NDV immunization 4 days later, NDV alone, or PBS control. Four weeks later, chickens in groups 1–3 received a booster NDV vaccination, while controls received PBS. Blood samples were collected at 4, 7, 14, and 21 days after primary immunization and 4 days after boosting. Splenic tissues were harvested at 4 days after both primary and booster immunization.

#### Antibody affinity measurement by ELISA

Serum antibodies were purified by caprylic acid–ammonium sulfate precipitation. Antibody affinity was determined using a solution-phase ELISA based on Friguet et al. (1985),^26^ in which purified antibodies were incubated with increasing concentrations of soluble IBDV VP2 or NDV F protein (generated in this study) until equilibrium was reached, followed by quantification of unbound antibody by indirect ELISA using HRP-conjugated goat anti-chicken IgG (Bioss, Cat#bs-0310G-HRP) and TMB substrate with absorbance measured at 450 nm. The equilibrium dissociation constant (Kd) was obtained by nonlinear regression in GraphPad Prism 9, and the affinity constant (Ka) was calculated as Ka = 1/Kd.

#### Hemagglutination (HA) assay

The hemagglutination (HA) titer of NDV antigen was determined to prepare 4 hemagglutinating units (HAU). NDV antigen was serially two-fold diluted in PBS (25 μL per well) in V-bottom 96-well plates, followed by addition of 25 μL of 1% chicken red blood cells and incubation at room temperature for 30 min. The HA titer (1 HAU) was defined as the highest dilution showing complete hemagglutination. The antigen was subsequently adjusted to 4 HAU for downstream assays.

#### Hemagglutination inhibition (HI) assay

Serum samples were collected at 7, 14, and 21 days after primary immunization and at 4 days after booster immunization. Blood was obtained from the wing vein, isolated by clotting at room temperature for 2 h followed by centrifugation (3000 rpm, 10 min, 4°C), and stored at −20°C until use. Serum samples were serially two-fold diluted in PBS (25 μL per well) and incubated with an equal volume of NDV antigen (4 HAU) at room temperature for 30 min. Chicken red blood cells (1%, 25 μL) were then added and incubated for an additional 30 min. HI titers were defined as the highest serum dilution that completely inhibited hemagglutination.

#### Serum IgM and IgY measurement by ELISA

Serum IgM and IgY levels were quantified using commercial ELISA kits (Abcam, IgM: Cat#ab157692; IgY: Cat#ab157693) according to the manufacturer’s instructions. Absorbance at 450 nm was measured and concentrations were calculated using a four-parameter logistic standard curve.

#### Preparation of splenic lymphocytes

Single-cell suspensions of splenic lymphocytes were prepared from chickens at 4 days after primary or booster immunization. Spleens were aseptically collected, mechanically dissociated through a 40 μm cell strainer in RPMI 1640 medium, and lymphocytes were isolated by density gradient centrifugation (2500 rpm, 10 min, room temperature). The interphase cells were collected, washed, and resuspended in PBS. Cells were counted and adjusted to the desired concentration.

#### Flow cytometry analysis

For staining, 2 × 10^6^ cells were incubated for 30 min on ice in the dark with mouse anti-chicken Bu-1-AF647 (SouthernBiotech, Cat#8395-31), IgM-FITC (Cat#8310-02), and IgY-PE (Cat#8320-09). Isotype controls included mouse IgG1-PE (Cat#0102-09), IgG2b-FITC (Cat#0104-02), and IgG1-AF647 (Cat#0102-31). After washing, cells were resuspended in PBS for acquisition. Data were acquired within 1 h using a FACSCalibur flow cytometer (BD Biosciences), with 20000 events collected per sample, and analyzed using FlowJo v10 software. Compensation was performed based on single-stained controls. Data analysis was conducted by gating on Bu-1^+^ B cells followed by quantification of IgM^+^ and IgY^+^ populations according to FITC and PE fluorescence signals.

#### Infection assays

DT40 cells were seeded at 1 × 10^6^ cells per well and infected with IBDV (MOI = 0.5) with or without NDV. After 2 h adsorption, cells were cultured in RPMI 1640 containing 2% fetal bovine serum, and RNA was collected at 6, 12, and 24 h post-infection for downstream analysis.

#### Overexpression assays

DT40 cells were transfected using an X-Porator H1 electroporator (BEX Co.) with plasmids encoding IBDV proteins (VP1–VP5) or control vector. Transfection was performed at 150 V with 4 pulses (630 ms interval, 1400 ms pulse width). Cells were cultured for 24 h prior to downstream analysis. Poly(I:C) (Sigma-Aldrich, Cat#P9582) was transfected under the same conditions.

#### qRT-PCR analysis

Total RNA was extracted from chicken spleen tissues or DT40 cells using the EASYspin Tissue/Cell RNA Rapid Extraction Kit (Aidlab, Cat#RN2801). For spleen samples, chickens were euthanized at 12 h after booster immunization. Spleens were collected, snap-frozen, and stored at −80°C. Approximately 50 mg of tissue was homogenized in lysis buffer, and RNA was isolated following standard procedures. For DT40 cells, total RNA was extracted at indicated time points after treatment or infection. cDNA was synthesized from 1 μg total RNA using HiScript IV All-in-One RT SuperMix (Vazyme, Cat#R433-01). Quantitative PCR was performed using Taq Pro Universal SYBR qPCR Master Mix (Vazyme, Cat#Q712) on a LightCycler 480 II system (Roche). Amplification was conducted with an initial denaturation at 95°C for 2 min, followed by 40 cycles of 95°C for 10 s and 60°C for 30 s. Gene expression levels were normalized to *GAPDH* using the ΔΔCt method. Primer sequences are listed in the Key Resources Table.

#### Western blot analysis

Cells were harvested 24 h after transfection and lysed in lysis buffer supplemented with protease inhibitor cocktail (MCE, Cat#HY-K0010). Proteins were separated by SDS-PAGE and transferred to PVDF membranes. Membranes were incubated with mouse monoclonal anti-Flag antibody (Sigma-Aldrich, Cat#B3111, 1:1000 dilution), followed by HRP-conjugated goat anti-mouse IgG (Dingguo Changsheng Biotechnology, Cat#SH-0011, 1:20000 dilution). Signals were detected using enhanced chemiluminescence.

### Quantification and statistical analysis

Statistical analyses were performed using GraphPad Prism 9. Data are presented as mean ± standard deviation (SD). Comparisons between groups were conducted using Student’s t-test for two groups or one-way ANOVA for multiple groups. Sample size (n) represents the number of biological replicates (animals or independent experiments), as indicated in the figure legends. No blinding was performed during data collection or analysis, and animals were randomly assigned to groups. Statistical significance was defined as ∗*p* < 0.05, ∗∗*p* < 0.01, and ∗∗∗*p* < 0.001.
